# Maintaining Brain Resilience with prevents disease in rat and mouse models of Alzheimer’s disease and restores brain health and cognition in chronic traumatic brain injury

**DOI:** 10.1002/alz.085726

**Published:** 2025-01-03

**Authors:** Andrew A. Pieper

**Affiliations:** ^1^ Case Western Reserve University, Cleveland, OH USA

## Abstract

**Background:**

Even when patients carry disease‐causing mutations their entire lives, they do not develop Alzheimer’s disease (AD) until later in life. The reason for this loss of brain resilience is not known, and two of the greatest risk factors for developing AD are aging and traumatic brain injury (TBI). Unfortunately, there are currently no protective treatments for patients that prevent the development of AD.

**Method:**

We assessed the ability of early treatment with the NAD^+^/NADH‐normalizing P7C3 compounds to prevent disease in the 5xFAD mouse and TgF344 AD rat amyloid‐based preclinical models of AD. We also assessed the therapeutic efficacy of delayed treatment with P7C3 compounds in a mouse model of chronic TBI. Lastly, we examined the ability of P7C3 compounds to protect human brain microvascular endothelial cells (HBMVECs) of the blood brain barrier (BBB) from oxidative stress, such as occurs in human AD.

**Result:**

Early administration of P7C3‐A20 to presymptomatic 5xFAD mice prevented plaque accumulation, tau phosphorylation, blood‐brain barrier deterioration, oxidative stress, DNA damage, neuroinflammation, impaired hippocampal neurogenesis, deficient synaptic plasticity, neurodegeneration, and neuropsychiatric and cognitive impairment. Similar protective efficacy was noted in TgF344 AD rats, and delayed treatment with P7C3 compounds in chronic TBI restored the BBB, arrested chronic neurodegeneration, and fully normalized cognitive function one year after injury. Lastly, treatment of HBMVECs with P7C3‐A20 protected them from the deleterious effects of exposure to otherwise overwhelming oxidative stress.

**Conclusion:**

Our results offer a first proof of principle for maintaining brain resilience to protect from developing AD, as well as the ability to reverse pathology and cognitive impairment in chronic TBI.

